# ID 336 - Dispositivos para Mensuração de Glicose em Pacientes Diabéticos: uma revisão sistemática de avaliações econômicas

**DOI:** 10.5327/2237-9622.2025.v34s1.336

**Published:** 2025-11-25

**Authors:** Andreia Ramos Lira, Adriane Lopes Medeiros Simone, Bruna Bento dos Santos, Stéfani Sousa Borges, Daniela Oliveira de Melo

**Keywords:** diabetes mellitus, revisão sistemática, avaliações econômicas em saúde, monitoramento glicêmico

## Abstract

**Introdução:**

As doenças crônicas não transmissíveis (DCNT), como o diabetes mellitus (DM), apresentam desafios crescentes à saúde pública e aos sistemas de saúde em termos de prevalência e de impacto econômico. No Brasil, mais de 16 milhões de adultos convivem com DM, demandando a implantação de estratégias para controle e cuidado eficazes. O controle da glicemia é essencial para reduzir complicações do DM, sendo o automonitoramento glicêmico (AMG) o mais utilizado, porém existem tecnologias mais modernas, como o monitoramento contínuo de glicose (MCG) e o monitoramento de glicose (MFG), que prometem otimizar o controle glicêmico, mas apresentam desafios de custo e implementação, particularmente no contexto do Sistema Único de Saúde (SUS). Assim, o objetivo desta revisão sistemática foi avaliar as evidências de Avaliações Econômicas em Saúde (AES) sobre dispositivos de MCG e MFG, explorando seus impactos e custos quando comparados ao AMG, considerando estudos primários de custo-efetividade, custo-utilidade e impacto orçamentário (AIO), sob perspectiva do SUS.

**Método:**

Foi realizada uma busca sistemática, em março de 2022, nas bases de dados MEDLINE (via PubMed), Embase (via Elsevier), Registry do Center for Evaluation of Value and Risk in Health (CEAR), Econlit, NHS Economic Evaluation Database, HTA Database (CRD), Epistemonikos e LILACS, além de repositórios brasileiros de teses e dissertações e manual em referências de publicações como forma complementar, para identificar AES completas que compararam dispositivos de monitoramento de glicose, sem restrição quanto à data de publicação. Dois revisores conduziram a seleção dos estudos de forma independente, resolvendo discrepâncias com um terceiro revisor.

**Resultados:**

Foram incluídos 23 estudos, sendo 18 AES e 5 AIO. A maioria das AES (77,78%) indicou que MCG e/ou MFG são custo-efetivos quando comparados ao AMG. Os estudos foram conduzidos principalmente em países desenvolvidos (83,33%) cujos limiares de disposição a pagar (WTP) variavam entre CAD 50.000 (Canadá) e £ 20.000 a £ 30.000 (Reino Unido) por QALY () e eram maiores que o estabelecido pela Comissão Nacional de Incorporação de Tecnologias no Sistema Único de Saúde (Conitec) de R$ 40.000 por QALY. Foram identificados conflitos de interesse e a presença de financiamento por fabricantes de dispositivos em 55,56% dos estudos, o que pode exercer influências sobre os resultados.

**Conclusão:**

Os dispositivos MCG e MFG demonstram oferecer melhorias clínicas e sociais importantes aos pacientes com DM, quando comparados ao AMG. Embora esses dispositivos sejam considerados custo-efetivos em países desenvolvidos com alto WTP, o limiar estabelecido pela Conitec sugere sua inviabilidade econômica no contexto do SUS. Apenas 44,44% dos estudos sugeriram que o MCG e o MFG eram custo-efetivos dentro do limiar brasileiro. Assim, a incorporação dessas tecnologias no SUS exige análise cuidadosa, desenvolvimento de estratégias que equilibrem os benefícios clínicos com as limitações orçamentárias do Brasil, além de uma compatibilização com os modelos de financiamento disponíveis.

